# Neural protein gamma-synuclein interacting with androgen receptor promotes human prostate cancer progression

**DOI:** 10.1186/1471-2407-12-593

**Published:** 2012-12-11

**Authors:** Junyi Chen, Li Jiao, Chuanliang Xu, Yongwei Yu, Zhensheng Zhang, Zheng Chang, Zhen Deng, Yinghao Sun

**Affiliations:** 1 Department of Urology, Changhai Hospital, Second Military Medical University, 168 Changhai Road, Shanghai 200433, China; 2 Department of Urology, the Second Affiliated Hospital of Fujian Medical University, Quanzhou, China; 3 Department of Pathology, Changhai Hospital, Second Military Medical University, Shanghai, China

**Keywords:** Prostate cancer, Gamma-synuclein, Androgen receptor, Metastasis

## Abstract

**Background:**

Gamma-synuclein (SNCG) has previously been demonstrated to be significantly correlated with metastatic malignancies; however, in-depth investigation of SNCG in prostate cancer is still lacking. In the present study, we evaluated the role of SNCG in prostate cancer progression and explored the underlying mechanisms.

**Methods:**

First, alteration of SNCG expression in LNCaP cell line to test the ability of SNCG on cellular properties in vitro and vivo whenever exposing with androgen or not. Subsequently, the Dual-luciferase reporter assays were performed to evaluate whether the role of SNCG in LNCaP is through AR signaling. Last, the association between SNCG and prostate cancer progression was assessed immunohistochemically using a series of human prostate tissues.

**Results:**

Silencing SNCG by siRNA in LNCaP cells contributes to the inhibition of cellular proliferation, the induction of cell-cycle arrest at the G1 phase, the suppression of cellular migration and invasion *in vitro*, as well as the decrease of tumor growth *in vivo* with the notable exception of castrated mice. Subsequently, mechanistic studies indicated that SNCG is a novel androgen receptor (AR) coactivator. It interacts with AR and promotes prostate cancer cellular growth and proliferation by activating AR transcription in an androgen-dependent manner. Finally, immunohistochemical analysis revealed that SNCG was almost undetectable in benign or androgen-independent tissues prostate lesions. The high expression of SNCG is correlated with peripheral and lymph node invasion.

**Conclusions:**

Our data suggest that SNCG may serve as a biomarker for predicting human prostate cancer progression and metastasis. It also may become as a novel target for biomedical therapy in advanced prostate cancer.

## Background

Prostate cancer (PCa) is the most commonly diagnosed malignancy and the second highest cause of cancer death in American men. Thus, PCa poses a major public health problem in the United States and worldwide [1]. In recent years, an upward trend in prostate cancer incidence has also been observed in Asian countries [2], possibly because of an increase in an aged population [3]. Although prostate-specific antigen (PSA)-based screening has become very common in the clinic, this marker lacks specificity [4]. Up to 25% of men with the disease have PSA levels less than 4.0 ng/ml, and “abnormal” or elevated PSA levels can also result from benign prostatic conditions [5]. A substantial proportion of screen-detected prostate cancers may have been overdiagnosed and subsequently overtreated, while others may not have been detected and treated early enough. The predictive value of conventional clinicopathological parameters for powerful prognosticators, such as pathological tumor stage and lymph node metastatic disease, remains limited. Widespread overtreatment has greatly increased the social burden and poor quality of life. Therefore, it is urgent to seek and refine prognostic information, which is gained from pretreatment variables and prostate cancer biopsy specimens in particular.

The synucleins are a small, soluble, highly conserved group of neuronal proteins that have been implicated in neurodegenerative diseases and cancer [6,7]. The synuclein family consists of α-, β-, and γ-synuclein (SNCG). The α- and β-synuclein proteins participate in the development and function of the central nervous system, and may be important in the etiology and pathogenesis of neurodegenerative disorders such as Alzheimer’s and Parkinson’s diseases [8-10]. SNCG is not clearly involved in neurodegenerative diseases. However, a stage-specific upregulation of SNCG has been found in advanced breast carcinomas [11] and other malignancies, including ovarian [12], gastric [13], esophagus [14], liver [15], colon [16], pancreatic [17], and bladder cancers [18]. In a pancreatic mouse model, SNCG emerged as the only upregulated molecule in a high perineural invasion group through proteomic and transcriptomic analysis [17]. Overexpression of SNCG interferes with drug-induced apoptotic responses [19] and mediates drug resistance [20]. Moreover, studies to date indicate that overexpression of SNCG compromises normal mitotic checkpoint controls, resulting in multi-nucleation and faster cell proliferation [21]. SNCG has been shown to promote cancer invasion and metastasis *in vitro* and in animal models [22]. There is a strong association between SNCG protein expression in primary tumors and distant metastases in multiple cancers. It has been implicated as a molecular indicator of metastasis in a wide range of human cancers [23]. Currently, there is no good biomarker for predicting the individual probability of metastatic progression of prostate cancer after radical prostatectomy. In this study, we explored if SNCG could serve as a biomarker for predicting human prostate cancer progression and metastasis.

## Methods

### Cell lines

The androgen-dependent human advanced prostate cancer cell line LNCaP was provided by Prof. Klaus Jung (Department of Urology, University Hospital Charité, Humboldt University, Germany). Androgen-independent PC-3 and DU145 cell lines were obtained from the Institution of Biochemistry and Cell Biology, the Chinese Academy of Sciences (Shanghai, China). The androgen-independent LNCaP (LNCaP-AI) cell subline was obtained from LNCaP cells cultured in androgen-deprivation medium as previously described [24].

### RNA interference

Small interfering oligonucleotides (oligo-166, 290 and 492) specifically targeting at human SNCG were synthesized and annealed by Genepharma Co, Ltd (Shanghai, China). The siRNA sequences were as follows: 5′-CCAUGGAUGUCUUCAAGAATT-3′ (forward) and 5′-UUCUUGAAGACAUCCAUGGTT-3′ (reverse) for oligo-166, 5′-CCAAGACCAAGGAGAAUGUTT-3′ (forward) and 5′-ACAUUCUCCUUGGUCUUGGTT-3′ (reverse) for oligo-290, 5′-GGUGAGGCAUCCAAAGAGATT-3′ (forward) and 5′-UCUCUUUGGAUGCCUCACCTT-3′ (reverse) for oligo-492. Negative control siRNA sequences were: 5′-UUCUCCGAACGUGUCACGUTT-3′ (forward) and 5′-ACGUGACACGUUCGGAGAATT-3′ (reverse).

### Establishment of stable SNCG cDNA-overexpressing and siRNA-expressing LNCaP cell lines

Full-length cDNA of SNCG gene (AF017256) was amplified from a plasmid, pGST-SNCG (a gift sent by Dr. Jia Zongchao in the Department of Biochemistry at the Queen’s University, Canada), and subcloned into a lentiviral vector pLV-RFP (Shanghai Invabio Bio-technology Co., China.) for construction of a lentiviral SNCG cDNA-overexpressing vector pLV-RFP-SNCG. siSNCG (oligo-166) or NC-negative was also constructed into a pLV-RFP vector. RFP-SNCG or RFP-siSNCG (oligo-166) vector was transfected into LNCaP cells. RFP empty vector or RFP-NC-negative control were induced in the same cells as the controls. After selection by puromycin treatment, an RFP positive clone was selected for utilization in the subsequent experiments. Transient transfection was used for cell culture experiments, and stably-transfected cells for some cell culture experiments and animal experiments. All of the experiments were performed three times and the results were reproducible.

### Quantitative RT-PCR

Total RNA was extracted with Trizol reagent (Invitrogen). Two micrograms of total RNA was used for the RT reaction (20 μl total volume) using the First-Strand cDNA synthesis kit (#K1621, Fermentas). One microliter of the cDNA was used as the template for quantitative PCR (SYBR Green _#_K0221, Fermentas), which was performed using the Lightcycler Detection System (Roche, Basel, Switzerland), according to the manufacturer’s instructions. The expression level of human glyceraldehyde 3-phosphate dehydrogenase (*GAPDH*) gene was used for normalization of SNCG mRNA expression level. The primers used in this study were 5′-CAAGAAGGGCTTCTCCATCGCCAAGG-3′ (forward) and 5′-CCTCTTTCTCTTTGGATGCCACACCC-3′ (reverse) for the human SNCG gene; 5′-TCTCAAGAGTTTGGATGGCTCC-3′ (forward) and 5′-TCACTGGGTGTGGAAATAGATG-3′ (reverse) for the human androgen receptor (AR) gene; 5′-TGGGAGTGCGAGAAGCATTC-3′ (forward) and 5′-GCACACAGCATGAACTTGGTCAC-3′ (reverse) for the human prostate specific antigen (PSA) gene; 5′-CGGAGTCAACGGATTTGGTCGTATTGG-3′ (forward) and 5′-GCTCCTGGAAGATGGTGATGGGATTTCC-3′ (reverse) for the GAPDH gene. Values represent the mean ± SD from at least three independent experiments, each performed in triplicate.

### Co-immunoprecipitation and western blot analyses

A co-immunoprecipitation assay was performed as previously described [25]. SNCG polyclonal antibodies (1:1000, sc-10699, Santa Cruz) and AR antibody (sc-815, Santa Cruz) were used for western blot and co-immunoprecipitation assay.

### Cell migration assay

Cell migration was measured using a Transwell chamber (Millipore, Germany). Briefly, RPMI 1640 medium containing 10% fetal bovine serum (FBS) was added into the lower compartment as a chemoattractant. After 24 h transfection, the cells were suspended in RPMI 1640 medium containing 1% FBS were seeded in the upper chamber and incubated for 20 hours at 37°C. The two chambers were separated by polycarbonate filters (8 μm pore size). At the end of incubation, cells on the top side of the filter were wiped off, and cells that migrated to the lower surface of the filter were fixed and stained with 0.1% crystal violet. Cell numbers were counted in five separate fields using light microscopy. The data were expressed as the mean value of cells in five fields based on three independent experiments.

### Cell invasion assays

Invasion assays were performed using 24-well Transwell units with 8 μm pore size polycarbonate inserts. The polycarbonate membranes were coated with Matrigel (Becton Dickinson) and cultured at 37°C for 1 h. After 24 h transfection, the cells (1.0 × 10^5^) were suspended in 200 μl of RPMI1640 medium containing 5% FBS and seeded in the upper compartment of the Transwell unit. Next, 500 μl of RPMI 1640 medium containing 10% FBS was added into the lower compartment as a chemoattractant. After 48 h incubation, cells on the upper side of the membrane were then removed, whereas the cells that migrated through the membrane to the underside were fixed and stained with 0.1% crystal violet. Cell numbers were counted in five separate fields using light microscopy. The data were expressed as the mean value of cells in five fields based on three independent experiments.

### Cell proliferation assays

Proliferation of LNCaP cells was evaluated by WST-8 Cell Counting Kit-8 (Beyotime, Jiangsu, China) assay according to the manufacturer’s instructions. This assay is based on the cleavage of the tetrazolium salt WST-8 by mitochondrial dehydrogenase in viable cells. Cells/well (1 × 10^3^) were incubated with 100 μl culture medium in 96-multiwell plates. Cells were cultured for 1, 2, 3, 5, and 7 days before addition of 10 μl CCK-8 to the culture medium in each well. After a further 4 h incubation period at 37°C, absorbance at 450 nm of each well was measured with a microplate reader (BioTek Instruments, Inc., USA). Each experiment was repeated three times, and the data represent the mean of all measurements.

### Cell cycle analysis

Cell cycle distribution was analyzed by flow cytometry. After the indicated treatments, cells were trypsinized, rinsed with PBS, and fixed with 70% ethanol at 4°C overnight. Fixed cells were washed with PBS and suspended in 500 μl of propidium iodide/Triton X-100/RNase staining solution for 30 minutes at 37°C in the dark. Cell cycle analysis was performed using a flow cytometer (MACSQuant™ Analyzer, Miltenyi Biotec). DNA histograms were analyzed by the MACSQuantify™ version 2.1.

### Dual luciferase reporter assays

Cells were transfected with 800 ng of a reporter plasmid pMMTV-LUC containing four different AREs. Then, 3 ng of a pRL-TK plasmid was also co-transfected as the internal control. After 24 h, the cells were treated with either ethanol or 1.0 nM DHT for 24 h. Luciferase assays were performed using the Promega Dual Luciferase Reporter Assay system.

### Tumorigenesis of human prostate cancer cells in nude male mice

Male athymic nude mice at 6–8-weeks-old were purchased from the Shanghai Cancer Institute, China. Animal handling and experimental procedures were approved by the Animal Investigation Committee of the Shanghai Cancer Institute. Tumors were generated by subcutaneous injection of 5 × 10^6^ siSNCG-166 and NC stably-transfected cells/mouse (n = 8 per group) mixed with 0.1 ml of Matrigel (BD Biosciences). The mice of the other two groups were castrated and then injected with stable SNCG cDNA-expressing LNCaP cells or RFP empty vector-expressing LNCaP cells as a control, and the tumors were measured twice weekly with a caliper. Tumor volume (cm^3^) was calculated by the formula ab^2^/2, where “a” was the largest diameter and “b” was the smallest diameter of the tumor.

### Tissue specimens and prostate tissue microarray (TMA)

Protocols involving human materials were approved by the institutional ethics committee of Shanghai Changhai Hospital, Shanghai, China. Formalin-fixed paraffin-embedded tissue specimens were obtained from the archives of the Department of Pathology. The specimens consisted of prostatitis tissues (n = 10), benign prostatic hyperplasia (BPH, n = 10), androgen-dependent prostate cancer (n = 122), and androgen-independent prostate cancer tissues (n = 5). Androgen-independent prostate cancer was defined as patients who become refractory after one to three years and resume growth despite hormone therapy. Tumors were staged following the standard Tumor-Node-Metastasis (TNM) methodology of American Joint Committee on Carcinoma (AJCC)/Union for International Cancer Control (UICC). This cohort of androgen-dependent prostate cancer patients did not receive neoadjuvant therapy such as radiation or hormonal therapy.

A prostate tissue microarray (TMA) was made from the formalin-fixed paraffin-embedded tissue specimens. Briefly, one core tissue-biopsy (diameter 0.6 mm) was taken from the marked region of individual paraffin-embedded prostate tumors and precisely arrayed into a new recipient paraffin block with a custom built precision instrument. Three TMAs containing an identical set of tumors were constructed. After the block construction was completed, 8- to 10-μm sections were cut with a microtome. The presence of tumor tissue on the arrayed samples was verified by H&E staining.

### Antibodies and immunohistochemical analysis

Goat anti-SNCG polyclonal antibody (sc-10699, Santa Cruz Biotechnology, CA) or rabbit anti-AR polyclonal antibody (sc-815, Santa Cruz Biotechnology, CA) were used for immunochemical staining by a standard ABC method. A semi-quantitative scoring system based on the average number of SNCG-positive cells from five randomly chosen × 400 fields was used to grade the expression levels. The mean value (n) was used to grade the expression levels: +, 0 < n ≤ 30; ++, 30 < n ≤ 50; +++, n > 50. Samples were independently evaluated under a light microscope by two pathologists without prior knowledge of the patients’ clinical data.

### Statistical analysis

All data were analyzed with SAS9.1.3 (SAS Institute, Cary, NC, USA). The Mann–Whitney Test was used to identify differences between SNCG protein expression and clinicopathologic features of prostate cancer. The Pearson’s correlation efficient analysis was applied across SNCG expression with AR status. The independent-samples *t*-test was analyzed for cell and animal experiments (mean ± SD). *P* < 0.05 was considered statistically significant.

## Results

### Silencing of SNCG by small-interfering oligonucleotides in LNCaP cells inhibits cellular proliferation and induces cell cycle arrest at G1 phase

**Figure 1:**
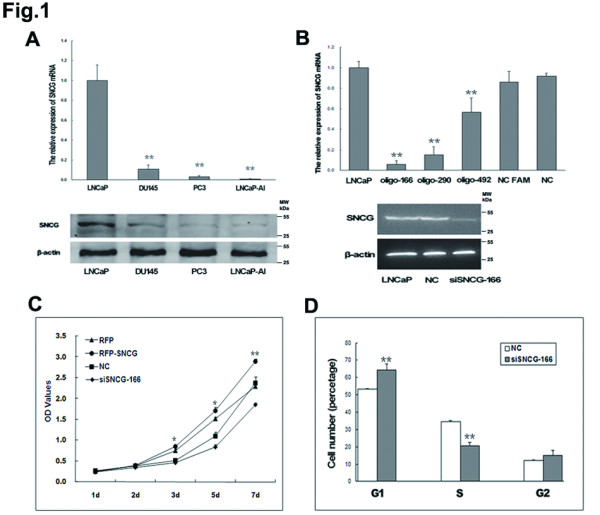
**Suppression of SNCG in LNCaP Cells inhibits cellular proliferation and induces cell cycle arrest at G1.** (**A**) **Top**: RT-PCR analysis was used to evaluate SNCG mRNA expression levels in androgen-dependent (LNCaP) and androgen-independent human advanced prostate cancer lines (DU145, PC3 and LNCaP-AI). **Bottom**: Western blot analysis revealed SNCG protein expression levels in the relevant cell lines. (**B**) **Top**: RT-PCR analysis was used to evaluate SNCG mRNA expression in LNCaP cells after transient transfection with various siRNA constructs. **Bottom**: Western blot analysis showed oligo-166 inhibited SNCG expression in LNCaP cells. (**C**) CCK-8 assay were performed for evaluation of the LNCaP cellular proliferation. (**D**) Flow cytometry assay demonstrated that inhibition SNCG expression in LNCaP cell induced cell cycle arrest at G1. These results are representative of three independent experiments. ^*^P < 0.05, ^**^P < 0.01.

To investigate SNCG expression patterns in prostate cancer, we first examined SNCG mRNA and protein expression levels in advanced human prostate cancer cell lines, including androgen-dependent LNCaP and androgen-independent DU145, PC3 and LNCaP-AI (androgen-independent LNCaP). LNCaP-AI cell subline was obtained from LNCaP cells cultured in androgen-deprivation medium. AR protein in LNCaP-AI is higher than in LNCaP, and LNCaP-AI cells showed stronger proliferative ability than LNCaP cells in androgen-deprivation culture medium. PSA secretion was stimulated with increasing concentrations of DHT in both LNCaP and LNCaP-AI cells, but the PSA secretion was much higher for LNCaP cells than for LNCaP-AI cells. RT-PCR was used to detect SNCG mRNA expression levels in total RNA samples extracted from four cell lines. The results showed that LNCaP cells expressed a high level of SNCG mRNA compared to DU145, PC3 and LNCaP-AI cells. Western blot analysis also revealed high levels of SNCG protein expression in LNCaP cells; however, low or undetectable levels of SNCG protein expression were found in DU145, PC3 and LNCaP-AI cells (Figure 1A).

To explore the effects of SNCG on prostate cancer cellular growth and proliferation, we employed RNA interference (RNAi) or full-length cDNA overexpression of SNCG gene in LNCaP cells. Three different SNCG-siRNAs and a negative control siRNA were transiently transfected into LNCaP cells to identify which siRNA sequence most potently suppressed SNCG mRNA levels. SNCG-siRNA significantly inhibited the relative expression of SNCG mRNA (oligo-166, 93% decrease; oligo-290, 84% decrease; and oligo-492, 42% decrease), whereas there was little difference between negative control siRNA and parental cells (Figure 1B). Oligo-166 was identified as the most potent suppressor of SNCG expression in LNCaP cells. Full-length SNCG cDNA sequence was sub-cloned into a pLeno-RFP retroviral vector. SNCG-RFP vector or effective RNA interference (siRNA) oligos was transiently transfected to LNCaP cells, and empty vector or nonsense RNA (NC) was transfected as the controls. A WST-8 Cell Counting Kit-8 (Beyotime, Jiangsu, China) assay was used for evaluation of cellular proliferation. RFP-labeled, SNCG-overexpressing LNCaP cells showed an increase in cellular proliferation compared to the control cells. As expected, silencing of SNCG in LNCaP cells (siSNCG-166) showed a decrease in cellular proliferation compared to the control cells (Figure 1C). To analyze the reasons for reduced cellular growth and proliferation in SNCG siRNA-expressing LNCaP cells, flow cytometry was used to examine cell cycle distribution. We observed 64.2% of siSNCG-166 cells were distributed in G1 phase, whereas only 53.4% of negative control cells remained in G1 phase (Figure 1D). Our results indicate that silencing of SNCG by siRNA in LNCaP cells suppresses cellular growth and proliferation and induces cell cycle arrest at G1 phase.

### Knockdown of SNCG by siRNA in LNCaP cells inhibits cellular migration and invasion *in vitro*

**Figure 2:**
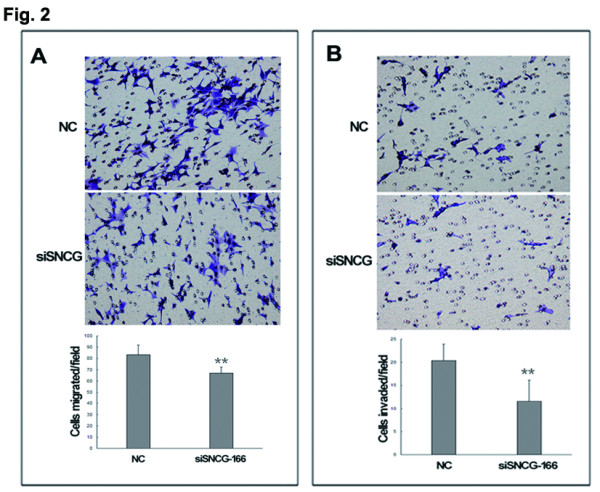
**Knockdown of SNCG by siRNA in LNCaP cells suppresses cellular migration and invasion *in vitro*.** (**A**) Inhibition of endogenous SNCG expression by siRNA reduced the number of cellular migration to the lower Transwell chamber normalized by the negative control. (**B**) SNCG knockdown decreased the number of cellular invasion by a Matrigel-Transwell assay compared to the negative control. Results are representative of three independent experiments. ^**^P < 0.01.

To investigate the relationship between SNCG expression and prostate cancer cellular biological behavior, we evaluated the effects of SNCG siRNA on cellular migration and invasion of LNCaP *in vitro* by chemotaxis and Matrigel invasion assays using Transwell chambers (Millipore, Germany). siSNCG-166 or nonsense RNA (NC) was transiently transfected into LNCaP cells (Figure 2). Chemotaxis or invasion through the Matrigel by siSNCG-166-expressing LNCaP cells was reduced by 20% or 43%, respectively, compared to the NC group. SNCG siRNA-expressing LNCaP cells showed a significant reduction in prostate cancer cellular migration and invasion compared to the control cells. The results suggest that silencing of SNCG expression by siRNA in LNCaP cells contributes to the suppression of cellular migration and invasion *in vitro*.

### SNCG protein interacts with androgen receptor in human prostate cancer cells

**Figure 3:**
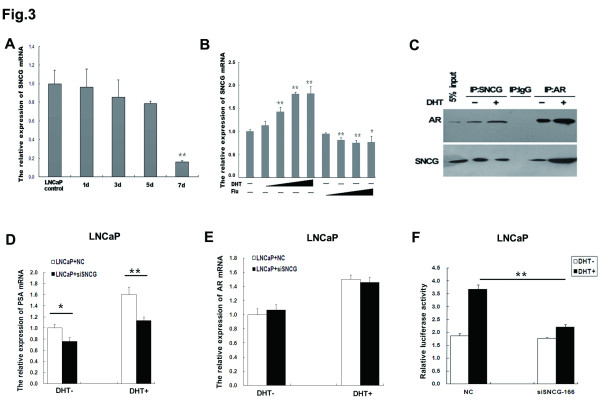
**Interaction between SNCG and AR protein regulates androgen**-**induced transcriptional activity of AR.** (**A**) After culture in androgen-deprived medium, SNCG mRNA expression in LNCaP cells was detected by RT-PCR. (**B**) LNCaP cells were treated with increasing amounts of androgens (dihydrotestosterone DHT at 0.1, 1.0, 10.0 and 100 nM) and anti-androgens (flutamide Flu at 0.1, 1.0, 10.0 and 100 μM) for 48 h before SNCG mRNA expression was analyzed by quantitative RT-PCR and compared to the levels in the parental LNCaP cells. (**C**) The co-IP assay indicated that the interaction between SNCG and AR was strengthened by DHT. IgG-precipitated complexes were used as the control. (**D**) PSA mRNA expression in siSNCG-LNCaP cells was measured after administration with androgen. (**E**) RT-PCR was used to detect the AR mRNA expression in siSNCG-LNCaP cells with or without DHT administration. (**F**) Dual-luciferase reporter assays were performed to show the AR transcriptional activity in siSNCG-LNCaP cells with or without DHT administration. ^*^P < 0.05, ^**^P < 0.01.

Since SNCG is expressed at high levels in androgen-dependent and at low levels in androgen-independent prostate cancer cells, we raised the question whether SNCG is involved in mediating hormone-dependent tumorigenicity. To test this, we investigated SNCG mRNA expression in LNCaP cells with or without androgen supplementation. After androgen deprivation, SNCG expression levels in LNCaP cells decreased with time (Figure 3A). To test whether the effects of dihydrotestosterone (DHT) administration on SNCG expression in LNCaP cells are mediated by androgen receptor (AR) signaling, we examined the effects of anti-androgen (flutamide, an AR antagonist) treatment on SNCG expression. Administration with anti-androgens mostly blocked DHT-induced SNCG expression, indicating that DHT modulates SNCG expression through AR signaling (Figure 3B).

To examine whether AR protein physiologically interacts with SNCG protein in human prostate cancer cells, we performed a co-immunoprecipitation assay. The lysates of LNCaP cells were immunoprecipitated with either an anti-AR or an anti-SNCG antibody. Then the membranes were immunoblotted with an anti-SNCG or an anti-AR antibody, respectively. We detected an interaction between AR and SNCG proteins in the lysates of SNCG-expressing LNCaP cells treated with or without DHT (Figure 3C), which was strengthened following DHT treatment. Under the same conditions, AR and SNCG proteins did not co-immunoprecipitate when the control IgG was used.

To further evaluate the relationship between SNCG and AR-mediated PSA expression, we examined whether altered SNCG expression in LNCaP cells results in changes in PSA transcription in response to DHT treatment. Knockdown of SNCG in LNCaP cells significantly reduced PSA mRNA expression induced by DHT, compared to the nonsense RNA control group (Figure 3D). We also examined AR expression levels in SNCG siRNA-expressing LNCaP cells. However, SNCG siRNA-expressing LNCaP cells had no significant effect on AR mRNA expression (Figure 3E). Then we examined the effects of SNCG on AR transcriptional activity by luciferase reporter assays. A plasmid containing androgen-responsive elements (AREs) was transfected into siSNCG-LNCaP cells or LNCaP cells transfected with nonsense RNA as the control. AR luciferase activity was significantly decreased with DHT treatment in SNCG siRNA group in contrast to the nonsense RNA group. These results suggest that SNCG is involved in androgen-induced AR transcriptional activity (Figure 3F).

These data indicated that SNCG, as a coregulator of AR, interact with AR protein and significantly affect AR target gene PSA expression by enhancing androgen-induced AR transcriptional activity.

### SNCG is involved in restoration of androgen sensitivity in LNCaP-AI cells

**Figure 4:**
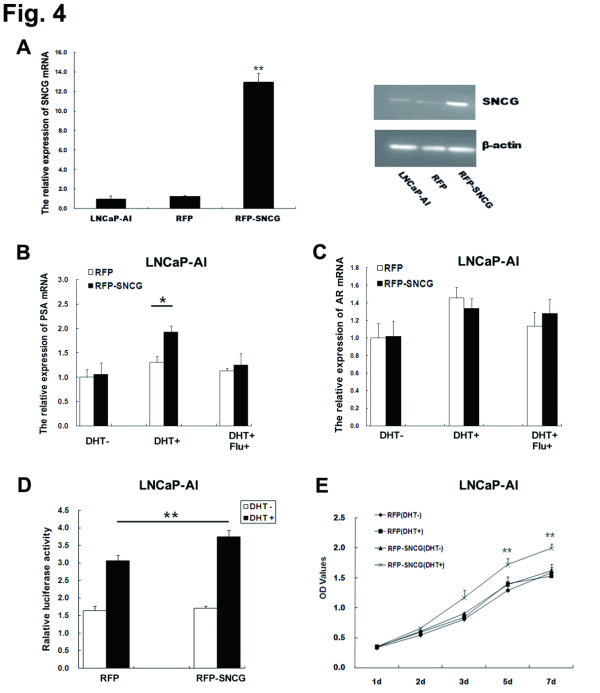
**Functional roles of SNCG in LNCaP**-**AI cells are ligand-dependent.** (**A**) LNCaP-AI cells were transfected with full-length SNCG cDNA plasmid or empty vector as the control and selected with puromycin treatment. A stable SNCG-overexpressing clone (RFP-SNCG) and a negative control clone (RFP) were used for the subsequent experiments. RT-PCR and western blot analysis showed the SNCG expression in RFP-SNCG, LNCaP-AI and RFP cells. (**B**) PSA mRNA expression in RFP-SNCG with androgen administration by RT-PCR compared to RFP. (**C**) RT-PCR was used to detect the AR mRNA expression in RFP-SNCG with or without DHT administration compared to RFP. (**D**) Dual-luciferase reporter assays were performed to show the AR transcriptional activity in LNCaP-AI cells with treatment of androgen. (**E**) The cellular proliferation was detected by CCK-8 assay in RFP-SNCG with or without DHT treatment compared to RFP. ^*^P < 0.05, ^**^P < 0.01.

Because of the observation that SNCG expression was regulated by androgen and was expressed a relatively low level in LNCaP-AI cells, we asked whether SNCG overexpression in LNCaP-AI cells contributes to androgen responsiveness. We first established a stable, RFP-labeled SNCG full-length cDNA-overexpressing LNCaP-AI cell line (Figure 4A), which was confirmed by fluorescence microscopy, RT-PCR and western blot. SNCG-overexpressing LNCaP-AI cells treated with DHT showed a significant increase in PSA mRNA expression compared to the control LNCaP-AI cells. The elevated PSA levels were blocked by flutamide treatment (Figure 4B). However, AR expression levels in LNCaP-AI cells were not affected by SNCG overexpression (Figure 4C). We found AREs activity detected by luciferase reporter assay in SNCG-overexpressing cells was significantly increased with DHT treatment compared to RFP vector-transfected control cells (Figure 4D). Additional DHT treatment did not significantly affect the proliferation rate of LNCaP-AI cells. However, SNCG-overexpressed LNCaP-AI cells showed an increase in cellular growth and proliferation in response to DHT treatment (Figure 4E), indicating that SNCG protein functions in affecting cellular growth response to DHT administration. Our data suggest that SNCG overexpression restores androgen sensitivity in LNCaP-AI cells via mediating AR transcription activity.

### SNCG promotes tumorigenesis of androgen-dependent prostate cancer cells *in vivo*

**Figure 5:**
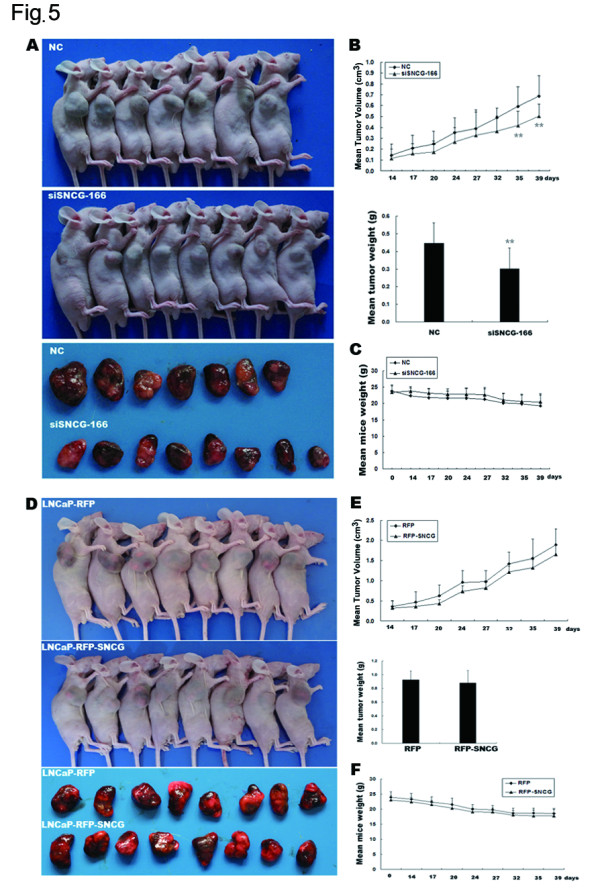
**SNCG promotes tumorigenesis of androgen-dependent prostate cancer cells.** (**A**) Tumorigenesis presented in siSNCG-166 group (n = 8) and NC group (n = 7, one mouse was sacrificed at 4 weeks after injection due to poor health) after subcutaneous injection of the cells. (**B**) **Top**: Tumor volume was observed twice weekly in the siSNCG-166 group compared to that in the NC group. **Bottom**: The mean tumor weight in the siSNCG-166 group was less than that in the NC group. (**C**): The mean mouse weight was measured twice weekly. No significant difference was observed between the siSNCG-166-LNCaP group and the NC-LNCaP group. (**D**) Mice were castrated before the indicated cells were injected. The tumor-bearing mice showed in RFP-SNCG-LNCaP group (n = 8) and RFP-LNCaP group (n = 8). (**E**) **Top**: Tumor volumes were observed twice weekly in RFP-SNCG-LNCaP group compared to RFP-LNCaP group. **Bottom**: The mean tumor weight showed in RFP-LNCaP group and RFP-SNCG-LNCaP group. (**F**): The mean mouse weight was measured twice weekly. No significant difference was observed between RFP-SNCG-LNCaP group and RFP-LNCaP group.

To investigate the effects of SNCG on LNCaP tumor growth *in vivo* associated with androgen status, we first analyzed tumorigenesis in response to androgen treatment in nude male mice. Tumors were monitored by caliper measurements (Figure 5A and B). Mice were imaged before being sacrificed. A significant delay in tumor growth was observed in the siSNCG-166 group compared to the NC group after 35 days, based on the analyses of gross tumor volume and weight and mouse body weight. A significant decrease in tumor weight was observed in the NC group compared to the siSNCG-166 group, indicating the importance of SNCG expression associated with LNCaP tumor growth *in vivo*. Next, we examined whether SNCG is involved in tumorigenesis of LNCaP cells with subcutaneous injection in castrated male nude mice. The mice were castrated after one week and were then injected with stable RFP-labeled SNCG-overexpressing LNCaP cells or RFP-expressing LNCaP cells as the control. There was no significant difference between two groups within 40 days post injection (Figure 5D and E), indicating that SNCG is involved in mediation of androgen-dependent prostatic tumorigenesis.

### SNCG protein expression is detected in human prostate cancer samples and correlates with clinicopathologic features of prostate cancer patients

**Figure 6:**
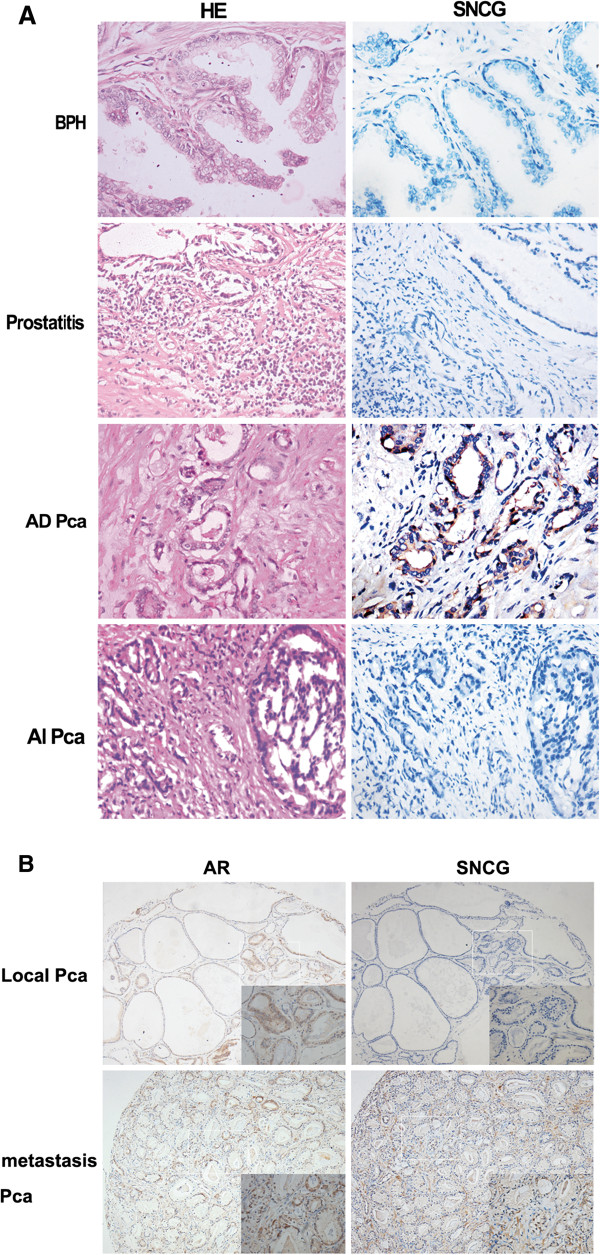
**SNCG protein expression is associated with human prostate cancer progression and metastasis.** (**A**) SNCG protein expression detected by immunohistochemical (IHC) staining was representative in a series of human prostate tissues on a tissue microarray (TMA). Benign prostatic hyperplasia tissues and prostatitis showed no SNCG expression in either epithelial or stromal regions. Strong staining of SNCG protein displayed in androgen-dependent prostate cancers and negative SNCG staining presented in androgen-independent prostate cancers. The left panel shows H&E staining and the right panel shows SNCG IHC staining (400×). (**B**) Representative immunohistochemical staining of SNCG (right line) or AR (light line) protein in local or metastatic human prostate cancer tissues.

To investigate the biological roles of SNCG in human prostate cancer progression and metastasis, an immunohistochemistry study was carried out on various tissue microarrays constructed with primary samples obtained from prostate cancer patients with known clinical and pathologic information by radical prostatectomy. SNCG protein was highly expressed in androgen-dependent (AD) prostate cancer cells but rarely expressed in benign tissues (BPH and prostatitis) or androgen-independent prostate cancer tissues (AIPC) (Figure 6). Statistically, prostate cancer tissues exhibited significantly higher SNCG expression than BPH and prostatitis with a *P* value of <0.001 (Table 1). To further investigate the correlation between SNCG protein expression and clinicopathological features, we evaluated the relationship between SNCG protein expression and PCa risk factors. SNCG expression levels were increased significantly in middle-high-risk PCa with PSA ≥10, Gleason score ≥7 and clinical stage ≥ T2b compared to low-risk PCa. Moreover, positive SNCG expression was more frequently detected in patients with lymph node metastasis (*P* < 0.001). Our data suggest that cytoplasmic expression of SNCG protein may serve as a prognostic biomarker to predict the occurrence of metastasis in advanced prostate cancer. However, there was no significant correlation between SNCG protein expression and AR state (r = 0.070, *P* = 0.881).

**Table 1:** Association between SNCG expression and clinicopathologic characteristics of patients with prostate cancer

		**SNCG**				**NO. samples**	**P**
		_**-**_	**+**	**++**	**+++**		
Prostatitis tissues		10	0	0	0	10	<0.001
BPH tissues		8	0	2	0	10	<0.001
Prostate cancer tissues (androgen-independent)		5	0	0	0	5	<0.001
Prostate cancer tissues (androgen-dependent)		48	24	31	19	122	
PSA	<10	22(57.9%)	9(23.7%)	6(15.8%)	1(3.6%)	38	0.004
	≥10	26(31.0%)	15(17.9%)	25(29.8%)	18(21.3%)	84	
Gleason score	≤6	19(59.3%)	6(18.8%)	5(15.6%)	2(6.3%)	32	0.037
	≥7	29(32.2%)	18(20.0%)	26(28.9%)	17(18.9%)	90	
Clinical stage	≤T2a	13(52.0%)	7(28.0%)	3(12.0%)	2(8.0%)	25	0.042
	≥T2b	35(36.1%)	17(17.5%)	28(28.9%)	17(17.5%)	97	
Lymph node invasion	Negative	46(42.2%)	24(22.0%)	28(25.7%)	11(10.1%)	109	<0.001
	positive	2(15.4%)	0	3(23.1%)	8(61.5%)	13	
AR	_-_	19(44.2%)	8(18.6%)	10(23.3%)	6(13.9%)	43	0.881
	_+_	29(36.7%)	16(20.3%)	21(26.6%)	13(16.4%)	79	

## Discussion

It has been demonstrated that SNCG is involved in tumorigenesis and metastasis of a wide range of malignancies [18-23]. SNCG-positive breast cancers have worse clinical outcome compared with SNCG-negative breast cancers. Only one report has documented a correlation between SNCG and prostate cancer metastasis [23]. The precise biological functions of SNCG and the mechanisms of its gene regulation in prostate cancer are still unknown. Metastasis is one of the hallmarks of advanced prostate cancer, and contributes to the high rates of morbidity and mortality in patients. In our studies, we found that SNCG expression in human prostate cancer cells results in a more malignant phenotype with increased cellular proliferation and motility *in vitro*. SNCG interacts with AR and enhances PSA expression mediated by androgen-induced transcriptional activity of AR. We also demonstrated that SNCG regulates androgen-dependent tumor size *in vivo*, and evaluated the clinical value of detection of SNCG protein expression in diagnosis of androgen-dependent prostate cancer.

To clarify the functional roles of SNCG in prostate cancer cells, we knocked down SNCG expression by siRNA in LNCaP cells and investigated its effects on cellular biological behaviors. Our data showed that silencing of SNCG in LNCaP cells contributes to suppression of cellular growth and proliferation, induction of cell cycle arrest at G1 phase and inhibition of cellular migration and invasion *in vitro*. These results are consistent with observations in many other human cancer cells and indicate that the functions of SNCG are not cell type-specific. The signaling pathways controlling SNCG gene regulation are still unknown. Some studies in other cancers reported SNCG is implicated in regulation of key steps of cellular proliferation, invasion and metastasis as well as survival. It may be activated through several cellular mechanisms, including reducing BubR1 protein levels [11,26,27], increasing ER-α transcription [28,29], activating RHO GTPase [30], MAPK and ElK1 [31], inducing MMP expression [32], and constitutive activation of ERK1/2 [19].

It was reported that SNCG expression was strongly correlated with the stages and Her-2 status [22,33]; however, this was not associated with ER and PR expression status in breast cancer studies [34]. We found SNCG expression is dependent on androgen status in human prostate cancer cells. Anti-androgen treatment mostly blocked DHT-induced SNCG expression, indicating that DHT modulates SNCG expression through AR signaling. This may account for our observations that SNCG expression was at an undetectable level in AIPC tissues and overexpression of SNCG did not affect tumorigenesis in the castrated male mice. To our knowledge, this is the first report that SNCG expression is dependent on androgen and plays an important role in prostate cancer progression. Consequently, SNCG may be closely associated with hormone-related tumors, and provide a new strategy for these tumors.

AR is a ligand-dependent transcription factor and a member of the class I subgroup of the nuclear receptor superfamily [35]. The androgen/AR signaling pathway is demonstrated to play a central role in prostate cancer development and progression. AR is activated by a ligand-dependent or a ligand-independent manner [36-38]. Subsequently, the activated receptor homodimerizes with AREs in the promoters of androgen target genes, resulting in activation of downstream gene expression [39]. Previous studies have demonstrated that AR signaling could be modulated by AR cofactors, such as heat-shock protein 27 [40], peroxiredoxin 1 [41], Tip60 [42], ARA54 [43], ARA55 [44], peroxisome proliferator-activated receptor coactivator-1 (PGC-1) [45] and human heterochromatin protein 1isoform HP1β [46]. SNCG has been shown to interact with ER and enhance ER transcriptional activity [29,30]. To explore whether SNCG is involved in mediation of AR signaling, we investigated the interaction between SNCG and AR proteins in LNCaP cells. Our results revealed that SNCG interacts with AR and its interaction is strengthened by DHT treatment. Although silencing of SNCG had no significant effect on AR expression in LNCaP cells, its suppression influenced PSA expression and AR transcriptional activity. We suggest that SNCG is a novel co-activator of AR and may play an important role in the molecular interaction with AR signaling in prostate cancer cells. The mechanisms need further exploration, including how they interact with each other, what downstream factor they promote or suppress, and so on.

Furthermore, we showed that silencing of SNCG by siRNA in LNCaP cells reduced tumor growth when the cells were injected into nude mice. These *in vivo* studies were consistent with the previously investigated functions of SNCG in prostate cancer cells *in vitro*. Our results indicate that aberrantly high expression of SNCG is partly responsible for tumor growth and invasion. Since SNCG expression of prostate cancer cells was regulated by androgen *in vitro*, we investigated stable SNCG-overexpressing LNCaP tumor growth in the castrated host mice. However, there was no significant difference between two groups with different expression levels of SNCG, indicating that SNCG regulates androgen-dependent prostate tumorigenesis. When prostate cancer patients are diagnosed at an advanced stage of the disease, androgen-deprivation therapy (ADT) has become the standard therapy. While the controversial topic remains, doctors believe that declining serum levels of testosterone and aging represent the most significant risk factors for prostate cancer progression [47-49]. A previous study claimed that exposure to reduced androgens may promote prostate tumorigenesis by activating special molecular events that drive more aggressive hormone-refractory tumors [50]. However, our data suggest that ADT therapy regimen in the treatment of advanced prostate cancer patients might effectively reduce some androgen-induced risk factors such as SNCG.

Abate-Shen showed that prostate tumors from low-testosterone mutant mice shared a similar gene expression profile to androgen-independent prostate tumors [50]. They suggested that declining serum levels of testosterone associated with aging is the main aggressive factor for prostate cancer. We raised the question whether it is necessary to perform androgen-deprivation (ADT) therapy on aged patients if they have high expression levels of SNCG protein. To address this issue, we overexpressed SNCG in androgen-independent LNCaP cells (LNCaP-AI). We found SNCG-overexpressing LNCaP-AI cells enhanced AR transcriptional activity and promoted PSA expression and cellular proliferation in response to DHT treatment. This suggested that SNCG may be a malignant risk factor in older men with prostate cancer.

Our results from a tissue microarray with immunohistochemical staining indicated SNCG protein is highly expressed in androgen-dependent (AD) prostate tumors, but is rarely expressed in benign tissues. In 122 radical prostatectomy specimens, 63.9% of SNCG-positive patients developed peripheral invasion. Only 20% of SNCG-high-positive (++ or +++) patients have local cancer. A total of 84.6% of SNCG-high positive (++ or +++) with lymph node metastasis indicate that SNCG expression is strongly associated with prostate cancer metastasis. The proportion of SNCG-positive metastasis is nearly consistent with previous reports [23]. Although SNCG protein expression was not associated with AR status, the high risk in malignant progression may help us establish efficient treatment strategies and reduce inappropriate or unnecessary treatments. SNCG expression was reported in 20% of preneoplastic lesions in the ovary [12], and was observed in various cancer types with lymph node invasion [23]. We found SNCG was not expressed in benign epithelium cells, but aberrantly expressed in advanced malignant states, suggesting that SNCG may be a tumor-oriented chaperone. The cases with high SNCG protein expression were found to be strongly associated with metastatic features. We therefore suggest SNCG protein expression in biopsy specimens may assist in distinguishing between potentially aggressive and potentially non-aggressive disease in prostate cancer screening. A large number of specimens need to be further explored to determine if SNCG protein expression is a predictive biomarker for evaluation of pre-surgery and survival of prostate cancer patients.

## Conclusions

In summary, SNCG is aberrantly expressed in PCa and is associated with its malignant progression. Our data provide evidence that SNCG protein expression may serve as a biomarker for assessment of biopsies to predict a high risk of prostate cancer progression and metastasis. SNCG is regulated by androgen, interacts with AR protein and affects AR target gene PSA expression by enhancing androgen-induced AR transcriptional activity, indicating that the functional roles of SNCG in PCa may be related to the androgen/AR signaling pathway. Because SNCG is involved in a variety of biological activities of PCa including cellular proliferation, migration and invasion *in vitro* as well as tumorigenesis *in vivo*, we suggest that modulation of SNCG expression might be a useful strategy for developing novel biomedical therapeutics for PCa.

## **The answers to the reviewers**

1. ***It would be preferable to see an additional AR dependent cell line e.g. CWR22 or use of the other siRNAs to see a dose response effect of SNCG inhibition.*** We detected the inhibition efficiency of oligo-166 target to SNCG in CWR22 cells, as expected, we had observed the inhibition effect of SNCG with dose dependent of siSNCG-166 in CWR22 cells. (Additional file 1: Figure S1).

2. ***The invasion/migration assays demonstrate a modest inhibition of invasion and migration, considering the level of SNCG knockdown, what is the mechanism for this inhibition? Is there an inhibition of MMP expression or Rho-GTPases for example?*** We supplement the experiment of the relation between inhibition SNCG and MMP using wild type LNCaP cells and stable cell line of RFP-SNCG-LNCaP and siSNCG-166-LNCaP (Additional file 2: Figure S2). From the result we confirmed the expression of SNCG is involving in the MMP members' regulation.

3. ***The authors looked at the effects of SNCG inhibition and increased expression in LnCap cells. Inhibition of SNCG resulted in decreased proliferation and accumulation in the G1 phase. Vice versa increased SNCG caused increased proliferation, however the authors do not present corresponding information on Cell cycle progression. Did they not collect this information?*** Some overexpression experiments were done later (Additional file 3: Figure S3), here we primarily show the role of inhibition of SNCG in LNCaP cells. In this way, it avoids the seeming confusion of the entire article structure.

4. ***The authors performed several experiments in LNCaP-AI cells. They should state how these cells compare to LNCaP cells in terms of AR levels, response to androgens, proliferation, etc.*** AR protein in LNCaP-AI is higher than LNCaP (Additional file 4: Figure S4). PSA secretion was stimulated with increasing concentration of DHT in both LNCaP and LNCaP-AI cells, but the PSA secretion was much higher for LNCaP cells than for LNCaP-AI cells. (Additional file 5: Figure S5).

5. ***The majority of the experiments rely on SNCG overexpression or knock-down but several of these experiments lack an important control: the authors should provide data showing SNCG protein levels for these experiments. The data should allow the reader to compare SNCG levels in control and over/underexpressing cells. This concerns the following figures: 1C-D, 2A-B, 3C-F and 5A-F.*** Transient transfection was used for cytological experiments. Before the cytological experiment (Figure 1- 3), we repeatedly proved a stable inhibition efficiency of SNCG by oligo-166 (in fact, more than 3 times). In Figure 1B, we show one of these experiments with protein expression. So, we did not show the protein expression in the every following experiment. The stably transfected cells screened by puromycin was used for animal experiments. We will supplement the fluorescence image of the cells and the protein level of the stable cell line of SNCG (Additional file 6: Figure S6). We have confirmed that SNCG protein expression in LNCaP cells, which were transiently transfected with SNCG plasmid or siRNA, was increased or decreased at different intervals up to 7 days (Additional file 7: Figure S7).

## Competing interests

The authors declare that they have no competing interests.

## Authors’ contributions

All the authors participated in processing the experiments of the data presented in this manuscript and/or verified the conclusions of this study. JC, ZC, and ZD carried out the experimental work. LJ, ZZ and CX provided clinical information and data analysis. YY provided tumor samples and histopathological analysis. YS, LJ, and JC conceived this study, made the constructs, participated in its design and coordination. All authors read and approved the manuscript.

## Pre-publication history

The pre-publication history for this paper can be accessed here:

http://www.biomedcentral.com/1471-2407/12/593/prepub

## Supplementary Material

Additional file 1: Figure S1 Western blot analysis was used to evaluate SNCG protein silence efficiency in CWR22 cells with different siSNCG-166 concentration. Click here for file

Additional file 2: Figure S2 Western blot analysis were used to detected the MMP members expression level with different SNCG expression state. Click here for file

Additional file 3: Figure S3 Cell cycles analyzed by flow cytometry. Up-regulation of SNCG expression in LNCaP promoted cells into S phase compared to REP-NC. Click here for file

Additional file 4: Figure S4 RT-PCR (A) and western blot (B) showed that the AR expression was higher in LNCaP-AI cells than in LNCaP cells **P<0.01. Click here for file

Additional file 5: Figure S5 Extracted from “Xu B, Sun Y, Tang G, Xu C, Wang L, Zhang Y, Ji J: Id-l expression in androgen-dependent prostate cancer is negatively regulated by androgen through androgen receptor. Cancer letters 2009, 278: 220-9”. Click here for file

Additional file 6: Figure S6 The stably transfected cell lines were constructed. The fluorescence image (up) and the protein level (down) were presented. Click here for file

Additional file 7: Figure S7 SNCG protein expression at indicated time points in LNCaP cells that were transfected with SNCG plasmid (A) or siRNA (B). Click here for file
