# Supplementary figures and images for: Neural protein gamma-synuclein interacting with androgen receptor promotes human prostate cancer progression
Source: BMC Cancer. 2012 Dec 11;12:593. doi: 10.1186/1471-2407-12-593 (PMC3599237; doi:10.1186/1471-2407-12-593)

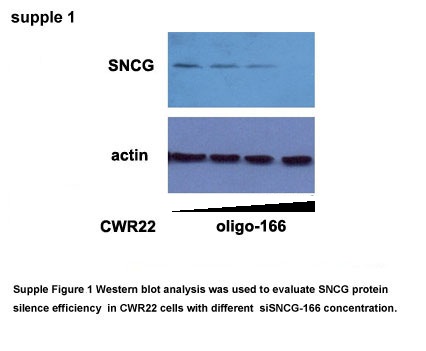

Supplement: Additional file 1: Figure S1 — Western blot analysis was used to evaluate SNCG protein silence efficiency in CWR22 cells with different siSNCG-166 concentration. [file 1471-2407-12-593-S1.jpeg]

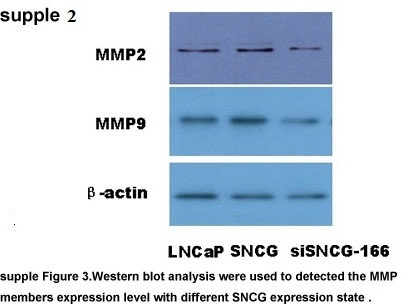

Supplement: Additional file 2: Figure S2 — Western blot analysis were used to detected the MMP members expression level with different SNCG expression state. [file 1471-2407-12-593-S2.jpeg]

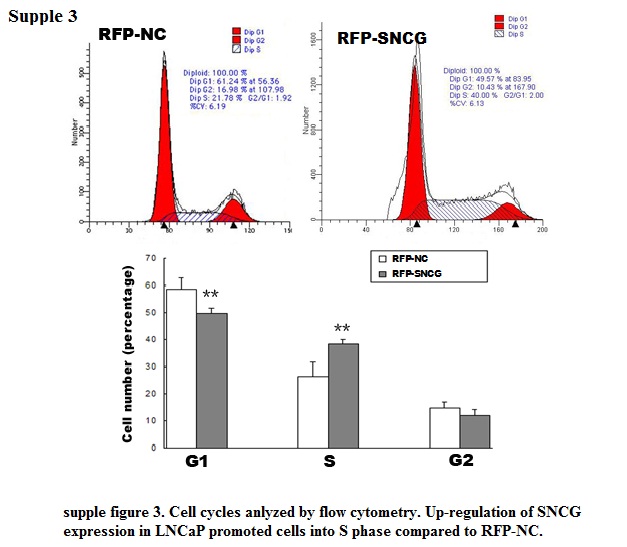

Supplement: Additional file 3: Figure S3 — Cell cycles analyzed by flow cytometry. Up-regulation of SNCG expression in LNCaP promoted cells into S phase compared to REP-NC. [file 1471-2407-12-593-S3.jpeg]

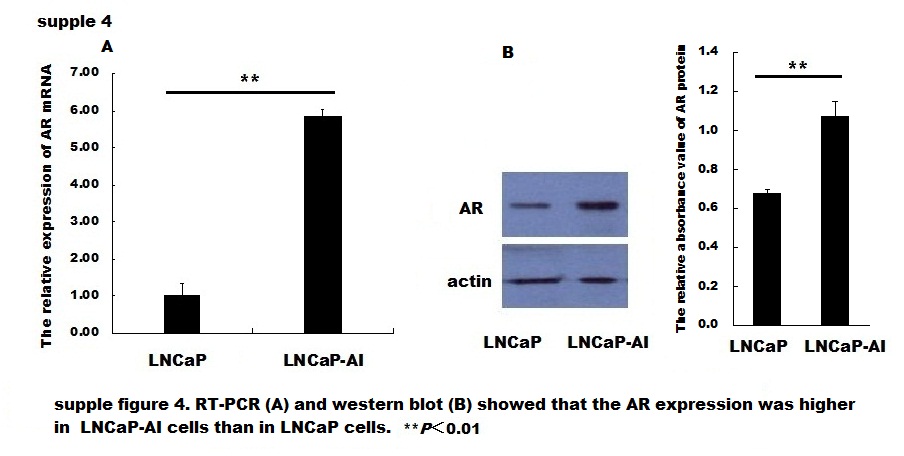

Supplement: Additional file 4: Figure S4 — RT-PCR (A) and western blot (B) showed that the AR expression was higher in LNCaP-AI cells than in LNCaP cells **P<0.01. [file 1471-2407-12-593-S4.jpeg]

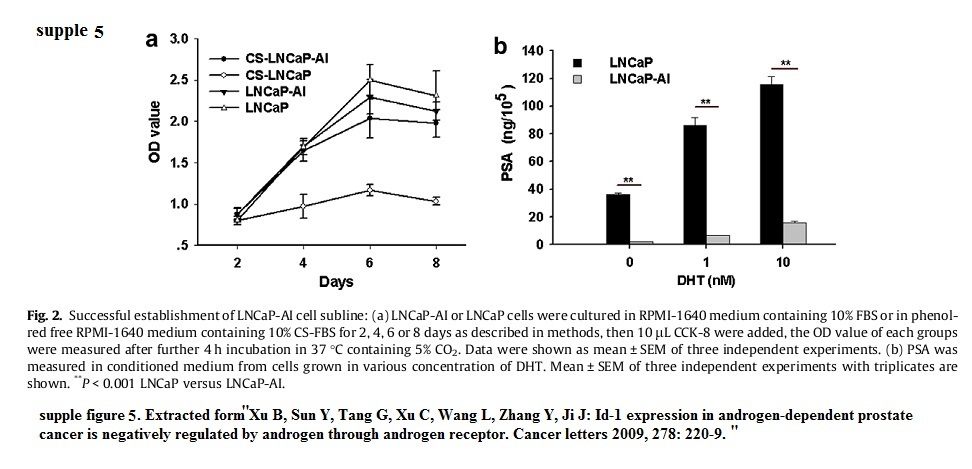

Supplement: Additional file 5: Figure S5 — Extracted from “Xu B, Sun Y, Tang G, Xu C, Wang L, Zhang Y, Ji J: Id-l expression in androgen-dependent prostate cancer is negatively regulated by androgen through androgen receptor. Cancer letters 2009, 278: 220-9”. [file 1471-2407-12-593-S5.jpeg]

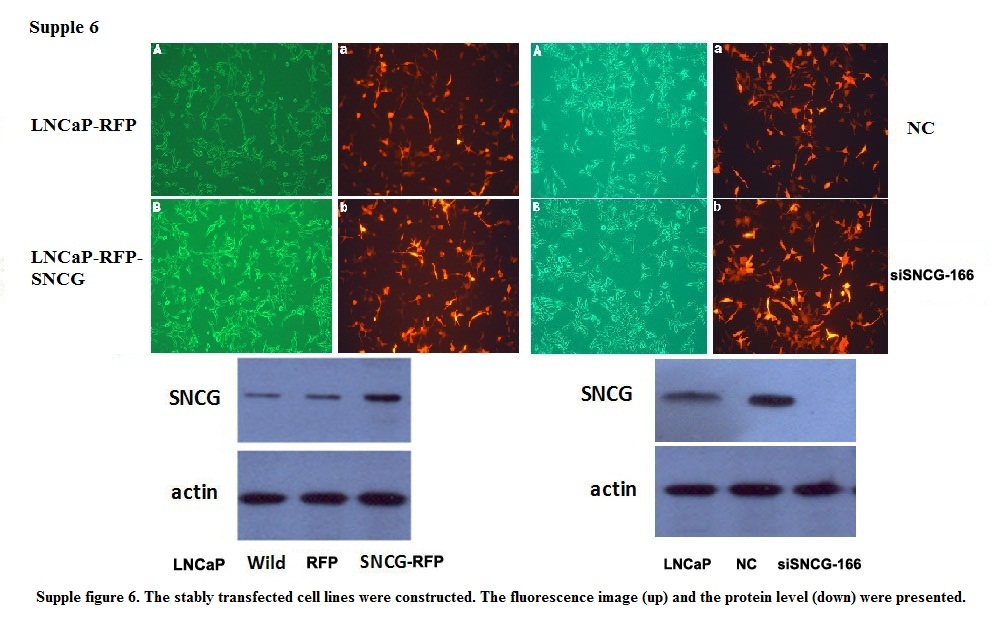

Supplement: Additional file 6: Figure S6 — The stably transfected cell lines were constructed. The fluorescence image (up) and the protein level (down) were presented. [file 1471-2407-12-593-S6.jpeg]

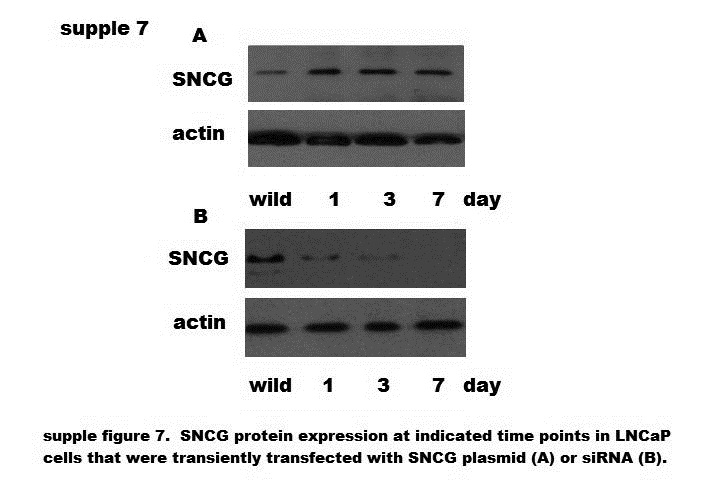

Supplement: Additional file 7: Figure S7 — SNCG protein expression at indicated time points in LNCaP cells that were transfected with SNCG plasmid (A) or siRNA (B). [file 1471-2407-12-593-S7.jpeg]
